# Early childhood caries: parents’ knowledge, attitude and practice towards its prevention in refugee camps in Erbil, Iraq

**DOI:** 10.1186/s12903-023-03516-8

**Published:** 2023-10-24

**Authors:** Hamsa Mohammed Al-Dahan, Sherzad Ali Ismael

**Affiliations:** 1 Dental Public Health, Kurdistan Board of Medical Specialties, Runaki, Erbil, Iraq; 2 Public Health Faculty, Kurdistan Board of Medical Specialties, Runaki, Erbil, Iraq

**Keywords:** Early childhood caries, Knowledge, Attitude, Practice, Refugees, Erbil, Iraq

## Abstract

**Background:**

Early childhood caries is a significant oral health issue in socially deprived communities, including refugees, where prevention plays a crucial role in minimizing the challenges and costs associated with treating early childhood caries. To improve oral health outcomes, it is important to understand parents’ knowledge, attitudes, and practices. This study aims to assess the oral health knowledge, practices, and attitudes of refugee parents.

**Methods:**

This cross-sectional study included 503 parents/caregivers residing in Erbil’s refugee camps in Iraq, with healthy preschool children aged one to six years. Structured questionnaire was utilized in conducting individual interviews with parents to evaluate their knowledge, attitudes, and practices. The questionnaire collected demographic information and data on access to oral health services.

**Results:**

A total of 503 out of 505 households actively participated in the study, resulting in a high response rate of 99.6%. Demographic analysis revealed that the majority of respondents were female parents, constituting 92.05% of the sample. Within the participant pool, the primary age groups were 26–35 years (55.3%) and 18–25 years (26.2%). Educational background analysis revealed that a significant proportion of parents had attained a secondary school education (29.6%) or primary school education (27.4%). Statistical analysis further established a noteworthy association between educational background and knowledge level. The investigation of participants’ knowledge uncovered notable gaps and misconceptions pertaining to early childhood caries, with an overall mean score of 5.1. Assessing the overall attitude of parents, a mean score of 3.87 (SD = 1.29) suggested a generally unfavorable attitude towards oral hygiene practices and prevention of early childhood caries. In terms of actual practices, parents demonstrated a mean practice score of 5.7.

**Conclusions:**

This study emphasizes knowledge gaps and misconceptions among parents in refugee camps regarding early childhood caries in preschool children. Findings revealed low knowledge scores, limited understanding of hidden sugars, delayed oral hygiene practices, and limited knowledge about fluoride.

## Introduction

Oral health is an essential part of the overall health and well-being of children. Poor oral health can affect children’s eating, speaking, learning, and socializing abilities. It can also lead to pain, infections, and tooth loss. Therefore, it is important to promote good oral health habits from an early age and prevent dental caries, which is the most common chronic disease of childhood. Dental caries is caused by the interaction of sugars from food and drinks with bacteria in tooth plaque, resulting in acid production that damages the tooth enamel. Dental caries can be prevented by regular tooth brushing with fluoride toothpaste, drinking fluoridated tap water, applying fluoride varnish or dental sealants to the teeth, and visiting the dentist regularly [1–5]. Socially disadvantaged populations, such as refugees, experience a higher prevalence and severity of Early Childhood Caries (ECC). Refugees face barriers related to awareness, access, affordability, and cultural appropriateness of dental services [6]. The Syrian conflict since 2011 has led to a significant refugee crisis, with over 5.5 million registered Syrian refugees, including 260,000 in Iraq, mostly residing in camps and informal settlements within the Kurdistan Region of Iraq [7]. Syrian refugee children in Iraq encounter various health challenges, including ECC, due to factors such as malnutrition, infectious diseases, and limited healthcare access [8]. Tooth decay during early childhood is influenced by multiple risk factors, including biological aspects such as nutrition, feeding practices, and early colonization of cariogenic microbes, as well as social factors like parental education, socioeconomic status, and knowledge about dental health [9, 10]. Treating ECC presents inherent challenges, including high costs, time consumption, and the need for specialized skills and experience in working with young children [11, 12]. Recurrence rates of ECC are significant, often requiring retreatment and potential rehabilitation under general anesthesia [13, 14]. Consequently, dental experts emphasize a preventive approach to ECC management [6, 15, 16]. Preventing ECC necessitates prenatal education, ongoing support for mothers and infants, dental care follow-up from an early age, and promoting good oral hygiene practices and fluoride application [17–21]. Oral health literacy plays a crucial role in overall well-being, and higher parental education and favorable socioeconomic status are associated with lower tooth decay rates in children [22–24]. Parental knowledge, attitude, and preventive practices significantly impact children’s oral health outcomes, underscoring the importance of parental understanding and involvement [25]. This study aims to assess the knowledge, attitude, and preventive practices of parents in Erbil refugee camps.

## Materials and methods

This is an observational cross-sectional study conducted among 505 parents residing in the four refugee camps in Erbil: Kawergosk, Darashakran, Basirma, and Qushtapa. The study included Syrian parents with healthy children aged 12 to 72 months, excluding those with mental and other developmental disorders. Out of 505 parents, 503 provided their consent to participate in this study.

Sampling was conducted using a multi-stage cluster sampling approach. Sampling sectors were randomly selected, and all residents within those sectors were included in the sample. Sample size for frequency in population size (for finite population correction factor or fpc)(N) is 5616, hypothesized % frequency of outcome factor in the population (p) is 80%+/-5, confidence limits as % of 100(absolute +/- %)(d) is 5%, design effect (for cluster surveys-DEFF) is 2, sample size(n) for various confidence levels (95%), the minimum sample size determined was 472. The following equation was used to determine sample size:

Sample size n = [DEFF*Np(1-p)]/ [(d2/Z21-α/2*(N-1) + p*(1-p)].

To ensure a convenient response rate, a total of 505 eligible households was taken. Open Epi, Version 3, the open-source calculator was used for sample size determination. Data collection occurred from October 2022 to February 2023, through individual interviews. Samples are taken proportionally to the total number of households in each camp.

In accordance with ethical considerations and regulatory guidelines, the research ethics committee of the Kurdistan Board of Medical Specialties has granted approval for the study protocol. Additionally, prior to participation in the study, informed consent was obtained from all parents or legal guardians of the participants. This ensured that participants’ rights and well-being were safeguarded throughout the research process. While the study is an observational cross-sectional design and does not involve experiments on humans or the use of human tissue samples, we have taken proactive measures to uphold ethical standards and maintain transparency in our methodology.

A modified questionnaire from a previous study in Malaysia was used in this study [26]. The questionnaire encompassed demographic information, knowledge, attitudes, and practices related to early childhood caries prevention, and access to services. The amendments included adding a question related to the socioeconomic/average income and adding a section to evaluate the access to services.To ensure accuracy, the questionnaire was translated into Arabic language and back-translated into English. The closed-ended questionnaire consisting of thirty items was employed, covering various factors related to early childhood oral health. The questionnaire which utilized different response formats encompassed three domains: knowledge, attitudes, and practices. Individual interviews ranged from 11 to 15 min.

Oral health and hygiene messages were delivered to the families who expressed interest in learning more. However, it’s important to note that some parents were extensively engaged in household tasks during the interview and were barely able to focus on filling out the questionnaire. Additionally, some were not interested or did not have the additional time to listen to the educational messages. These messages encompassed information on various topics, including the transmission of bacteria from caregivers to babies, appropriate methods and timing for cleaning babies’ gums and teeth, the proper use of fluoridated toothpaste, and dietary habits related to dental health.

Data analysis involved the use of a scoring system to evaluate participant responses. A score of 1 was assigned for correct answers, while incorrect answers received a score of 0 in the knowledge, attitude, and practice sections. Individual scores were summed to obtain a total score for each section, ranging from 0 to 9. These scores were further categorized as good, medium, or poor. Scores of 7–9 were considered good, indicating high knowledge, positive attitude, and effective practices. Scores of 4–6 were categorized as medium, representing moderate levels. Scores of 1–3 were classified as poor, indicating low knowledge, negative attitude, and inadequate practices.

Data analysis was performed using SPSS version 25.0. ANOVA, Chi-square, and descriptive statistics were employed to examine the relationships between variables and parents’ knowledge, attitude, and practice. Descriptive statistics were used to calculate response frequencies and percentages for each question. The significance level was set at 0.05, and the P-value was calculated with a 95% confidence interval.

## Results

Among the 505 parents or households invited to participate in the study, an impressive response rate of 99.6% was achieved, with 503 parents ultimately taking part.

**Table 1:** Demographic data of the parents

Participants’ demographic data		Frequency	Percentage
Sex	Female	463	92.05
	Male	40	7.95
Status	Single	0	0.00
	Married	499	99.20
	Divorced	3	0.60
	Widower	1	0.20
Age	18–25	132	26.24
	26–35	278	55.27
	36–45	88	17.50
	46–49	3	0.60
	50+	2	0.40
Educational background	Never been in school	61	12.13
	Primary school	138	27.44
	Secondary school	149	29.62
	High School	110	21.87
	Diploma	21	4.17
	Bachelor	24	4.77
What is your average income in Iraqi Dinners (ID)	Less than 500,000 ID	414	82.31
	500,000–1,000,000 ID	33	6.56
	No income	53	10.54
	Refused to answer	3	0.60

Table 1 provides an overview of the demographic characteristics of the participating parents, revealing that the majority were females, constituting 92% (n = 463), while males accounted for 8% (n = 40) of the sample. The majority of the parents fell within the age range of 26–35 years (55.3%), followed by those aged 18–25 years (26.2%). Regarding educational attainment, a considerable proportion of parents had completed secondary school (29.6%) or primary school (27.4%). Regarding family income, the majority of participants (82.3%) reported an income of less than 500,000 ID, while (6.6%) reported an income between 500,000–1,000,000 ID. Additionally, 10.5% reported having no income, and a negligible proportion (0.6%) declined to provide income information.

**Table 2:** Frequency distribution of knowledge of the parents

Knowledge Section	Answer	Frequency	Percentage
Tooth Decay Can affect infants below 2 years of age	True	285	56.66
	False	155	30.82
	Don’t Know	63	12.52
When does the first baby tooth appear in the child’s mouth?	6-12months	482	95.83
	after the age of 24 m	10	1.99
	Don’t Know	11	2.19
Your Child will have a complete set of 20 milk teeth by the age of	30-36months	227	45.13
	12-18 m	138	27.44
	Don’t Know	138	27.44
The main type of food that can cause tooth decay are	Sugar, carbs	476	94.63%
	Other	7	1.39%
	Don’t Know	20	3.98%
Weaning from a baby bottle to a sipping cup should be planned when the child is	6-12months	257	51.09%
	after the age of 24 months	208	41.35%
	Don’t Know	26	5.17%
Cleaning your baby’s mouth after each meal should begin even before teeth erupt	others: depending on the child’s desire	12	2.39%
	True	98	19.48%
	False	340	67.59%
Brushing your baby’s teeth is important for preventing tooth decay	True	484	96.22%
	False	7	1.39%
	Don’t Know	12	2.39%
Fluoride in toothpaste is important for preventing tooth decay	True	78	15.51%
	False	24	4.77%
	Don’t Know	401	79.72%
When you should start using toothpaste with fluoride for cleaning your child’s teeth?	1 year	11	2.19%
	Other (school age, after the eruption of permanent teeth)	103	20.48%
	Don’t Know	389	77.34%
It is necessary to do fillings in the baby’s teeth	True	174	34.59%
	False	271	53.88%
	Don’t Know	58	11.53%

Table 2 displays the parents’ responses to the knowledge questions. The average knowledge score among parents was 5.14 out of 10 (SD = 1.248).

The study found that parents had some basic knowledge about their children’s dental health, but also had gaps and misconceptions. For instance, 95.8% knew the correct age range for the first baby tooth, but only 45.1% knew when a child would have a full set of 20 teeth. About 56.66% were aware that tooth decay could affect infants below two years old, and 94.6% identified the main food causing tooth decay. However, knowledge regarding the appropriate time to stop bottle feeding was low (51.09% mentioned 6–12 months). While 96.2% recognized the importance of brushing a baby’s teeth, only 19.4% knew to clean the mouth after each meal even before teeth erupt. Many parents lacked information about fluoride in toothpaste (84.4%) and only 2.19% knew when to start using fluoride toothpaste. Moreover, only 34.5% recognized the need for filling cavities in baby teeth.

Table 3 summarizes the parents’ attitudes toward various factors related to oral health in early childhood. The overall mean attitude score was 3.87 (SD = 1.29), indicating a generally poor attitude towards oral hygiene practices and ECC prevention.

Regarding specific attitudes, only 36% of parents believed that tooth decay can be caused by bacteria transmitted through sharing feeding utensils. A very small percentage (0.4% and 3.5%) mentioned that children should visit the dentist after 6 months and 1 year of tooth eruption, respectively. A majority (52.9%) believed it should be during school age, while a significant proportion (37.6%) did not know when to take their baby for a dental check-up.

About 48.9% of parents thought that nighttime bottle/breastfeeding does not cause tooth decay, and 55% believed that frequent and prolonged daytime breast/bottle feeding does not cause tooth decay. Only 31.6% agreed that a child’s teeth should be cleaned/brushed as soon as they erupt. Approximately half of the parents (50.3%) agreed that effective teeth cleaning can be achieved by the child themselves.

In terms of fluoride, only 41.7% understood its harmful effects when swallowed, while 32% did not know what fluoride was or its potential consequences if swallowed. A majority (70%) were against visiting the dentist before the age of two, and 83.5% recognized the negative impact of prolonged pacifier use on the normal development of children’s teeth.

**Table 4:** Frequency distribution of practices of the respondents

Practice Section	Answer	Frequency	Percentage
Do you bite the food into small pieces before giving it to your child	Always	3	0.60
	Often	24	4.77
	Sometimes	62	12.33
	Never	414	82.31
How often do you examine the mouth of your baby	Always	23	4.57
	Often	102	20.28
	Sometimes	337	67.00
	Never	41	8.15
How often do you buy sweetened food for your child?	Always	171	34.00
	Often	117	23.26
	Sometimes	188	37.38
	Never	27	5.37
How often do you give sweetened liquid/juice to your baby in a bottle	Always	38	7.55
	Often	129	25.65
	Sometimes	106	21.07
	Never	230	45.73
How often do you give plain water after each feed?	Always	72	14.31
	Often	201	39.96
	Sometimes	147	29.22
	Never	83	16.50
When did you start semisolid food for your child?	After the age of 6 m	465	92.45
	before the age of 6 m	37	7.36
	Don’t know	1	0.20
How often do you brush your Baby’s teeth/day	Always	32	6.36%
	Often	58	11.53%
	Sometimes	159	31.61%
	Never	254	50.50%
How much toothpaste do you use to brush your child’s teeth?	Pea size	90	17.89%
	Rice size	112	22.27%
	Brush length	3	0.60%
	Never	298	59.24%
Did you use a pacifier dipped into a sweet liquid for your child	Always	10	1.99%
	Often	22	4.37%
	Sometimes	56	11.13%
	Never	207	41.15%
Do you take an effort to improve your dental health knowledge	Always	4	0.80%
	Often	90	17.89%
	Sometimes	236	46.92%
	Never	173	34.39%

Table 4 displays the parents’ behaviors related to oral health in early childhood.

The overall mean score for parents was calculated to be 5.78. The majority of parents (91.8%) have examined their children’s mouths, while most parents (82.3%) have never bitten food into small pieces before giving it to their child. A small percentage of parents (5.3%) reported never buying sweetened food for their children.

Less than half of the parents (45.7%) reported never offering a bottle of sweetened juice to their babies. The majority of parents (83.5%) practiced giving their child plain water after meals. Similarly, 92.4% started introducing semisolids to their child after the age of six months. A small percentage of parents (17.5%) admitted to dipping the pacifier into a sweet liquid for their child.

Parents were almost evenly divided between those who brushed their children’s teeth (49.5%) and those who never brushed their children’s teeth (50.5%). Among those who practiced brushing, only 6.3% did it twice daily. When asked about the amount of toothpaste used, 17.8%, 22.2%, and 0.6% mentioned using a pea size, rice size, and toothbrush length, respectively.

Finally, only 34.3% of parents indicated that they have never tried to improve their dental health knowledge.

**Fig. 1:**
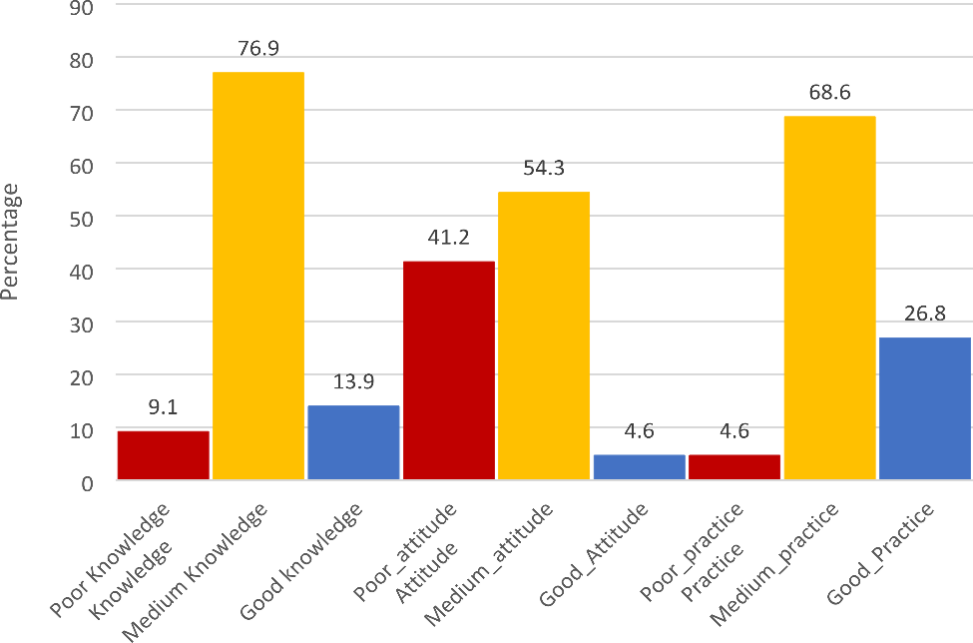
Distribution of parents’ scores by knowledge, attitude, and practice categories

Figure 1 illustrates the scores of parents categorized by their knowledge, attitude, and practice levels. The results reveal that the majority of parents exhibit a medium level of knowledge (76.9%) regarding the prevention of early childhood caries (ECC), whereas a smaller proportion demonstrates poor (9.1%) or good (13.9%) knowledge. In terms of attitude, a significant percentage of parents display a poor attitude (41.2%), while the majority hold a medium attitude (54.3%) and a minority exhibit a good attitude (4.6%). Concerning practice, the majority of parents engage in medium-level practices (68.6%), with a noteworthy percentage demonstrating good practice (26.8%) and a small fraction displaying poor practice (4.6%). These findings shed light on the need for targeted interventions to enhance parents’ knowledge, attitude, and practices related to ECC prevention, thereby promoting oral health among preschool children in refugee camps.

Table 5 displays the relationship between various independent variables and the dependent variables: Knowledge, Attitude, and Practice. The independent variables include Gender, Educational Background, and Socioeconomic status/Average Income.

Regarding Gender, the analysis suggests that there were no statistically significant differences in Knowledge (p = 0.149) and Attitude (p = 0.111) between male and female participants. However, in terms of Practice, the difference was not statistically significant (p = 0.466). Both genders demonstrated comparable levels of knowledge, with female participants showing slightly higher scores.

In terms of Educational Background, there was a statistically significant relationship between the level of education and Knowledge, Attitude, and Practice (p < 0.001). Participants who had never been to school had significantly lower scores compared to those with varying levels of formal education. The pattern indicates a progressive increase in Knowledge, Attitude, and Practice scores with higher levels of education.

Examining the association with Socioeconomic status/Average Income, the data revealed a significant association with Knowledge (p = 0.002), while no significant association was found for Attitude (p = 0.385) and Practice (p = 0.296). Participants with a lower average income had lower levels of Knowledge compared to those with higher incomes. However, socioeconomic status did not appear to significantly influence Attitude and Practice.

**Table 5:** Association between the dependent and independent variables

Independent Variables		Frequency	Dependent Variables		
			Knowledge Mean ± SD (P-value)	Attitude Mean ± SD (P-value)	Practice Mean ± SD (P-value)
Gender	Male	40	5.12 ± 0.822 (0.149)*	3.53 ± 1.154 (0.111)*	5.93 ± 1.18 (0.466)*
	Female	463	5.14 ± 1.278	3.90 ± 1.449	5.77 ± 1.311
Educational Background	Never been in school	61	(< 0.001)**	(< 0.001)**	(< 0.001)**
	Primary school	138			
	secondary school	149			
	High School	110			
	Diploma	21			
	Bachelor	24			
Socioeconomic status/Average Income	Less than 500,000 ID	414	(0.002)**	(0.385)**	(0.296)**
	500,000–1,000,000 ID	33			
	No income	53			
	Refused to answer	3			

*Results were calculated using ANOVA test

** Results were calculated using Fisher’s exact test

In terms of access to dental health services, a significant proportion of the participants (77%) reported having access to such services. Among those with access, the majority (59.3%) utilized private clinics, while governmental services were utilized by 25.8% of the participants, and 13.1% reported using NGO clinics. However, only a minimal percentage (1.8%) reported receiving health education related to dental health.

Regarding the frequency of dental clinic visits among those with access to services, the majority of participants (73.4%) reported visiting the clinic only when necessary. A small percentage reported regular visits every six months (1.0%), annual visits (0.2%), or never visiting the clinic (2.6%).

When queried about their exposure to dental health education and information regarding dental hygiene practices to maintain oral health, the majority of participants (94.2%) indicated a lack of receipt of dental health education. Only a single individual (0.2%) reported receiving a brochure, two participants (0.4%) received information from both a doctor/dentist and a brochure, twelve participants (2.4%) obtained information solely from a doctor/dentist, three participants (0.6%) received information from health promoters, and eleven participants (2.2%) received information from TV or social media sources.

## Discussion

This study aimed to assess the knowledge, attitude, and preventive practices of parents regarding early childhood caries (ECC), residing in refugee camps in Erbil city. The results revealed a low mean dental knowledge score among the parents (5.1), which aligns with similar findings from previous studies conducted in the Middle East and other countries [27–29], but contrasts with findings from other investigations [26, 30].

The mean knowledge score for females was 5.14, while for males it was 5.12, and no significant difference was found between the two groups. This contrasts with previous studies where mothers tended to score higher in the knowledge Sects. [29, 31]. The discrepancy observed in our study might be attributed to a lack of parental education regarding oral health at the camp level. In terms of attitude, the mean score was 3.8, indicating a less favorable attitude among the parents. Interestingly, the mean practice score was 5.7, suggesting that the parents’ actual practices were more positive than their knowledge and attitude. A similar trend was observed in a previous study [32], where the knowledge score was 6.95, the attitude score was 3.35, and the “good” and “bad” practice scores were 3.35 and 1.85, respectively. Additionally, another study [33] reported that parents exhibited adequate knowledge about oral health, but their actual practices fell short.

The majority of parents participating in this study demonstrated awareness of the association between sugar consumption and tooth decay; however, their dietary practices for their children did not align with this knowledge. According to the survey findings, 94.6% of parents correctly identified the main type of food that can lead to tooth decay, yet the same percentage admitted to purchasing sweetened food for their children on a daily basis or at least three times a week. Additionally, 54.2% of parents provided their children with juice or sweetened liquids in bottles, and 17.5% disclosed that they dipped pacifiers into sweet liquids. These findings are consistent with previous studies that have identified similar gaps in parental knowledge and behavior concerning sugar consumption and its association with tooth decay [26, 28]. Nonetheless, the survey did reveal some positive dietary practices among parents, including introducing semi-solid foods to babies after the age of six months (92.4%) and offering plain water after meals (83.5%).

Feeding practices have been found to impact the risk of early childhood caries (ECC) in children, as highlighted in a previous study [34]. Continuing to nurse beyond 12 months, frequent nighttime breastfeeding after tooth eruption, and allowing a baby to sleep with a bottle containing fermentable carbohydrates can increase the risk of ECC. However, our study revealed that many parents are unaware of these risk factors and do not follow healthy feeding habits. In our sample, 81% of parents were not aware of the association between nighttime feeding and tooth decay, 85.4% were unaware of the link between frequent and prolonged daytime feeding and tooth decay, and 51% believed that transitioning from bottles to sipping cups should occur between 6 and 12 months of age. These findings align with a previous study conducted in Saudi Arabia [28] but differ from a study in Malaysia [26] where a higher proportion of parents demonstrated knowledge of appropriate feeding practices.

Early oral hygiene practices are crucial for children, starting before teeth erupt and continuing with tooth brushing once the first tooth appears. The American Academy of Pediatric Dentistry recommends brushing at least once daily until the age of 2, and then increasing to twice daily [35]. Research has shown that early initiation of tooth brushing reduces the incidence of tooth decay [36]. However, our study found that only 19.4% of parents were aware of the need to clean a baby’s mouth before tooth eruption, despite 96.2% recognizing the importance of tooth brushing for preventing decay. Furthermore, only 31.6% of parents agreed to clean their child’s teeth as soon as they erupt, and half of the parents never practiced tooth cleaning. Only a small percentage (6.3% and (11.5%) reported cleaning their child’s teeth twice and once a day, respectively. Surprisingly, 50.3% of parents believed children could effectively clean their own teeth. These findings differ significantly from a previous study [26] where 100% of parents acknowledged the importance of tooth brushing, 81% recognized the need for pre-eruption cleaning, 88% agreed on cleaning teeth immediately after eruption, and only 2% never brushed their baby’s teeth. These results underscore a substantial gap in knowledge, attitudes, and practices among parents regarding the oral hygiene of their children in this population.

The significance of fluoride in strengthening enamel and preventing tooth decay has been well established [26]. However, our study revealed a lack of awareness among 79% of parents regarding the role of fluoride in caries prevention, with 4.7% expressing disagreement. Furthermore, 41.7% of parents believed that swallowing fluoride toothpaste could harm their child’s health. In terms of toothpaste usage, only 17.8% and 22.2% of parents utilized pea-size and smear-size amounts for their children, respectively, while 59.2% never used toothpaste at all. These findings differ from a previous study conducted in Malaysia [26], where parents displayed adequate knowledge about oral health and used fluoride toothpaste for their children.

The vertical transmission of oral microorganisms through activities like kissing and sharing utensils is a key focus in oral health education. However, our study reveals concerning gaps in knowledge among participants. Specifically, 41.9% disagreed with the idea that bacteria can be transmitted through shared feeding utensils, and 22% were unsure about this possibility. Interestingly, despite this limited knowledge, a significant majority of parents (82.3%) reported never biting hard food into small pieces before giving it to their children. This suggests that certain feeding practices may be inherited or influenced by cultural traditions rather than solely based on scientific knowledge. In contrast, a previous study [26] reported a different pattern, with 67.6% of parents practicing food biting for their children. These discrepancies underscore the potential variations in cultural practices and parental behaviors related to feeding habits across different populations. They also emphasize the influence of cultural norms on oral health behaviors and highlight the importance of considering contextual factors when developing oral health interventions.

The recommended timing for children’s first dental visit is within 6 to 12 months after the eruption of their first primary tooth [37]. However, this study reveals a significant lack of awareness and disagreement among parents. 52% believed that their child should visit the dentist during school age, while 37.5% were uncertain. Only 3.5% suggested a dental visit at one year of age, 0.4% after six months, and 5.5% stated they would never take their child to the dentist. Notably, the majority (70.3%) did not consider it necessary to visit the dentist before the age of two years. These responses may stem from the misconception that primary teeth hold less importance than permanent teeth. Similar findings were reported in another study [38] where 32% of mothers would only seek dental care for their children in the event of toothache. Moreover, 46% indicated a dental visit at one year of age, 17% at the eruption of the first primary tooth, and 4% mentioned visiting the dentist when the child is just one month old. Although 77% of parents reported having access to dental health services, the majority (95%) only sought such services when needed, with a mere 1.6% reporting routine checkups.

Targeted oral health education programs in refugee camps are crucial for improving children’s oral health outcomes. These programs should prioritize educating parents about hidden sugars, fluoride benefits and risks, appropriate feeding practices, and early dental visits. Cultural factors and unique challenges must be considered to ensure culturally sensitive interventions. Future research should assess the effectiveness of culturally tailored oral health education programs in ECC prevention, explore the impact of social support networks and access to oral health services, track children’s oral health outcomes over time, compare knowledge and practices across different camp settings, and cultural backgrounds, and utilize qualitative methods to understand cultural influences on parental behaviors.

### Limitations

The study has a limitation concerning the approach utilized to evaluate knowledge, attitudes, and practices. As highlighted by [39], this specific method of assessment may inherently lack accuracy. When participants are interviewed face-to-face by a professional, there is a possibility that they might offer responses based on what they believe they should know, rather than providing a true reflection of their actual behaviors or practices. This phenomenon can introduce bias into the data and might not yield a completely reliable representation of participants’ genuine knowledge, attitudes, and behaviors.

Another limitation of this study is its limited generalizability. This study focused on parents from four refugee camps in Erbil city. While this multi-camp approach allows for a broader representation of the refugee population in this region, it is important to acknowledge that the findings may still be specific to the Erbil context and may not necessarily apply to refugees in other regions or countries. Additionally, it’s essential to note that camp residents have been confined to the camps since 2014 and may have had limited interaction with the host community or refugees who have resided in urban areas. Such circumstances may have influenced their knowledge, attitudes, and behaviors differently from refugees in other settings.

## Conclusions

This study highlights gaps and misconceptions among parents in refugee camps regarding early childhood caries (ECC) in preschool children. Low knowledge scores, lack of understanding about hidden sugars, delayed oral hygiene practices, and limited knowledge about fluoride were observed. Targeted education programs are crucial to promote early oral hygiene, healthier diets, proper use of fluoride, and timely dental visits. Cultural considerations should be taken into account. By improving parental knowledge and practices, ECC prevalence and severity can be reduced, benefiting the oral health of preschool children in refugee camps. Therefore, there is a crucial need to educate parents about the impact of feeding practices on oral health and promote the adoption of healthier habits to enhance the well-being of their children.

**Table 3:** Frequency distribution of attitude of parents

Attitude Section	Answer	Frequency	Percentage %
Tooth decay is caused by bacteria that are transmitted by sharing feeding utensils (e.g., spoon)	Strongly agree	35	6.96
	Agree	146	29.03
	Don’t know	111	22.07
	Disagree	158	31.41
	Strongly disagree	53	10.54
When do you think you should take your baby for a dental check-up after the teeth erupt?	6 months	2	0.40
	1 year	18	3.58
	Don’t know	189	37.57
	School-age	266	52.88
	Never	28	5.57
A balanced diet is essential for the healthy growth of a baby’s teeth	Strongly agree	206	40.95
	Agree	267	53.08
	Don’t know	11	2.19
	Disagree	12	2.39
	Strongly disagree	7	1.39
Night-time bottle /breastfeeding can cause tooth decay	Strongly agree	20	3.98
	Agree	75	14.91
	Don’t know	162	32.21
	Disagree	195	38.77
	Strongly disagree	51	10.14
Frequent and prolonged breast/bottle feeding in the daytime can cause tooth decay	Strongly agree	7	1.39
	Agree	66	13.12
	Don’t know	153	30.42
	Disagree	224	44.53
	Strongly disagree	53	10.54
A child’s teeth should be cleaned/ brushed as soon as the teeth erupt	Strongly agree	12	2.39
	Agree	147	29.22
	Don’t know	60	11.93
	Disagree	267	53.08
	Strongly disagree	17	3.38
Effective cleaning of the teeth can be achieved by the child him/herself	Strongly agree	11	2.19
	Agree	242	48.11
	Don’t know	15	2.98
	disagree	205	40.76
	Strongly disagree	30	5.96
Swallowing of fluoride toothpaste can be harmful to a child’s health	Strongly agree	29	5.77
	Agree	181	35.98
	Don’t know	161	32.00
	Disagree	124	24.65
	Strongly disagree	8	1.59
It is important for a child to visit the dentist before the age of 2 years	Strongly agree	2	0.40%
	Agree	79	15.71%
	Don’t know	68	13.52%
	Disagree	329	65.41%
	Strongly disagree	25	4.97%
Prolonged use of a pacifier can affect the normal development of a child’s teeth	Strongly agree	149	29.62
	Agree	271	53.88
	Don’t know	39	7.75
	Disagree	32	6.36%
	Strongly disagree	12	2.39%

## Data Availability

All data used and analyzed in this study are available upon request. Interested parties seeking access to the data from this study are kindly requested to contact the corresponding author, Dr. Sherzad. Your inquiries and requests for data can be directed to [sherzad.aismael@gmail.com]. We are committed to facilitating transparent and responsible data sharing to promote scientific collaboration and further advancements in the field of public health.
